# Primary school learners’ movement during class time: perceptions of educators in the Western Cape, South Africa

**DOI:** 10.1186/s12889-023-17428-3

**Published:** 2023-12-13

**Authors:** Dominic Fisher, Quinette Louw

**Affiliations:** Faculty of Medicine and Health Sciences, Department of Rehabilitation and Health Sciences, Division of Physiotherapy, Stellenbosch University, PO Box 241, Cape Town, 8000 South Africa

**Keywords:** Sedentary Behaviour, Class time movement, Classroom furniture, Teacher-related factors

## Abstract

**Background:**

Over the last decade, the efficacy of in-classroom movement interventions to reduce sedentary behaviour has been mainly conducted in high-income, developed countries. To date, there have been no published reports on the perceptions of principals and teachers regarding learners’ movement during class time to inform the implementation of classroom-based movement strategies to reduce sedentariness in South Africa.

**Methods:**

A sample of primary school principals and grades 5 and 6 teachers from a range of socio-economic categories participated in this exploratory, descriptive qualitative study in the metro central district of the Western Cape Education Department in South Africa. Transcripts of individual depth interviews (IDIs) with principals and focus group discussions (FGDs) with teachers were coded using Atlast ti 9* software for qualitative analysis.

**Results:**

Thirteen principals (13 IDIs) and 24 teachers (6 FGDs) participated in the study. Two main factors influencing in-classroom movement, namely teacher-related and structural factors, were identified. The teacher-related factors pertained to their classroom management practice, knowledge and beliefs about sitting, and management style influenced their role in delivering the curriculum and creating a classroom environment conducive for learning. Classroom factors pertained to classroom size, the number of learners in the class and the ergonomic utility of traditional classroom desks. Institutional expectations and acceptance of learners’ behaviour, and teachers’ practice were also notable contributing factors that influenced learners’ movement during class time.

**Conclusion:**

Emerging evidence about the efficacy of in-classroom movement on sedentary behaviour and learning outcomes challenges traditional teacher practice that sitting is essential for the delivery of the curriculum and for creating an optimal learning environment. Teacher training about the behaviour control utility of in-classroom movement, and school management and policy supportive of in-classroom movement may encourage fidelity to in-classroom movement strategies.

**Supplementary Information:**

The online version contains supplementary material available at 10.1186/s12889-023-17428-3.

## Background

Children spend most of their time at school being sedentary with sitting being the predominant sedentary behaviour [1]. Sedentary behaviour is defined as waking behaviour that involves energy expenditure less than or equal to 1.5 metabolic unit equivalents (METs) while in sitting, reclining, or lying down [2]. Uninterrupted sitting has been shown to result in appreciable short-term unwanted metabolic changes [3] while prolonged sedentary behaviour is a risk for developing chronic non-communicable diseases [4]. In addition, prolonged sitting has also been shown to increase axial loading of the spine and increased back muscle activation which could lead to back pain [5]. Prolonged static sitting has also been shown to negatively affect concentration [6]. Since sedentary behaviour [7] and back pain [8] both track from childhood to adulthood, prevention of these conditions should be targeted in young populations. Schools are therefore a strategic setting in which to promote healthy behaviour to mitigate the risk of preventable health conditions.

Several studies set in the school environment have investigated the effectiveness of interventions aimed at reducing learners’ sitting time [9, 10] by creating a more dynamic classroom environment. Flexible classrooms provide learners with alternatives to traditional desks, namely, couches, ottomans and raised writing surfaces and don’t have a distinct ‘front’ with smart boards and whiteboards available around the room [11]. Although effective, the feasibility of the stealth strategy of the flexible classroom approach to increase learners’ movement during class time remains untested. Interrupting prolonged bouts of in-classroom sitting with short movement breaks have shown to reduce sitting time [12–15]. An increasingly popular strategy to increase classroom dynamism and reduce classroom sitting is by replacing traditional classroom desks with sit-stand desks [9, 15–17]. Sit-stand desks facilitate an easy transition between sitting and standing during class time. Learners may be encouraged to choose sitting over standing or teachers may dictate their postural topography. Generally, interventions have an educational component that informs the participants about the importance of increasing their activity to negate the potential harms of prolonged static sitting. These strategies to reduce sitting time and increase the amount of movement during class time and regularly change learners’ body position may potentially improve learners’ metabolic and musculoskeletal health [6] and improve their attention [18]. However, the changes to the classroom environment and the additional responsibility to facilitate classroom-based movement programmes are likely to affect teachers’ usual practice.

Published reports of teachers’ perspectives of various in-classroom movement programmes have mainly been conducted in developed countries such as the USA [19], UK [20], Canada [21], Ireland [22], and Slovenia [23]. In general, these studies report a relatively positive attitude, and a wide-range in-classroom movement strategies being used by teachers. Although teachers in these studies acknowledge the benefits of in-classroom movement on learners’ physical activity and engagement with learning activities amongst others, they also report potential barriers, such as a lack of time and the competing demands of the curriculum [24]. However, generalising findings from published reports conducted in vastly different socio-economic contexts and applying them to developing, middle income contexts such as South Africa, should be done cautiously. With the success of interventions dependent on teachers’ participation, understanding their perspectives of the prevailing factors that influence learners’ movement during class time is a necessary step prior to attempting to implement formal movement integration strategies such as those referred to previously.

There is a paucity of research on this subject in the South African context. A recent qualitative study conducted in SA reported on the perception that sitting duration has an influence on the onset of spinal pain [25]. An unpublished report showed that teachers are mainly unaware of the effects of prolonged classroom sitting on learners’ health [26]. Although the study recommends further research into the feasibility and acceptability of classroom-based-interventions to address these issues, there is a lack of understanding of the factors that influence learners’ movement during class time in SA schools. Given that teachers are the gatekeepers of the classroom environment and primarily responsible for the behaviour of children, it is important to understand their perspectives on learners’ movement in the classroom [6]. To our knowledge there are no published reports about educators’ perspectives on the factors that influence learners’ movement during class time, including the role they may play, if any.

As one of the most unequal countries in the world [27], persistent income polarisation in South Africa results in chronic poverty. Most of the population live below the national poverty line with more than one third living below the international poverty line of USD 3.20 per person per day. Furthermore, South Africa faces a quadruple burden of disease from communicable diseases (such as HIV/AIDS and TB), maternal and child mortality, NCDs (such as hypertension and cardiometabolic disease), and injury and trauma [28]. Public health and education system budgets are severely constrained by the country’s socio-economic difficulties. South Africa also continues to deal with the effects of social segregation perpetrated by the decades long Apartheid policy. The new National Health Insurance Bill [29] prioritises better linkages between education and health departments to drive prevention policies and initiatives. The country has severe shortages of teachers so posts cannot be filled and consequently classes are large, especially in schools situated in previously disadvantages areas. Local research that informs preventative behaviours and initiatives in schools may contribute towards better health of young people. The aim of this study was to explore South African educators’ perspectives and factors that influence learners’ movement and sitting patterns during class time.

## Methods

The Consolidated Criteria for Reporting Qualitative Research (COREQ) [30] statement guided the methodology and reporting of this study. Ethical approval for this study was obtained from the Health Research Ethic committee of Stellenbosch University (S17/08/130) and the permission was obtained from the Western Cape Education Department (WCED) (Reference 20170525-1279).

### Study design

An exploratory, descriptive qualitative study design based on individual depth interviews (IDIs) and focus groups discussion (FGDs) to understand the factors that influence learners’ in-classroom movement and positioning was used.

### Setting

The study was conducted in the metro central school district of the WCED between August 2017 and February 2018. Schools in South Africa are categorised using the National Quintile System [31], ranking from quintile one to five. The quintile system is based on the income, literacy, and unemployment levels of the surrounding community. Quintile one is the lowest socio-economic quintile, while quintile five depicts the highest socio-economic quintile. Most schools in the metro central district are categorised as quintile five (49.4%), followed by quintile four (35.3%), quintile three (10.9%) and quintile two (1.3%) with no quintile one schools. Although the metro central district is a highly populous, predominantly urban region and has the most primary schools of all the districts in the WCED, it may not be representative of rural (farm) schools. The metro central district was selected for pragmatic reasons given its proximity to the research team and is representative of the urban districts with the WCED.

### Sampling and recruitment

Most school-going children in South Africa attend publicly funded schools. An estimated 1% of children attend privately funded schools. The sampling framework using the National Quintile System was used to stratify publicly funded primary schools in the metro central district. This sampling strategy allows for greater variety in the sample [30]. Independent and special education needs schools were excluded from the sample. Independent schools are governed by a different regulatory authority than public schools and generally have greater access to resources relative to the number of learners enrolled. Special education needs schools are often adapted to the needs of the learners resulting in greater variation between schools and are thus very different to the predominant public-school context. Schools in each quintile strata were randomly selected to be contacted telephonically or via email for the purposes of recruitment into the study. If there was no response from the school (email or telephonically), no further contact was made.

**Fig. 1:**
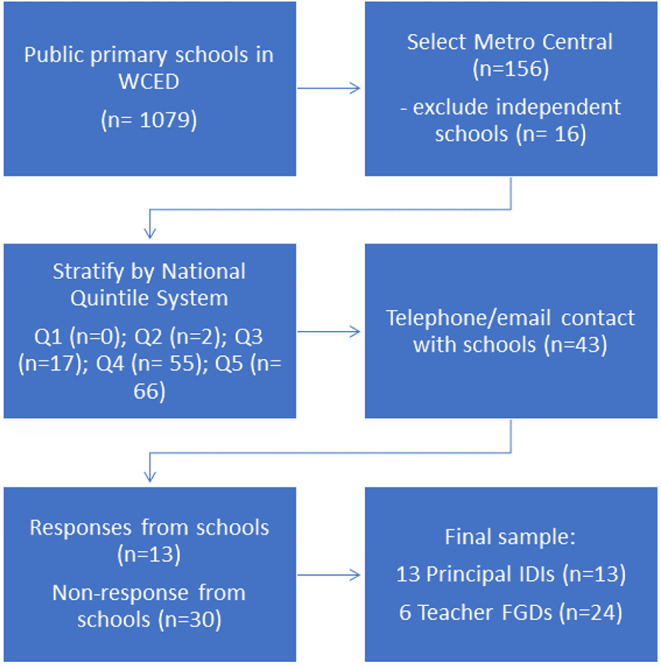
Sample and recruitment strategy

School principals who showed interest in participating in the study were emailed project information materials (Appendix 1) and consent forms (Appendix 2) explaining the nature and purpose of the study and were invited to participate in the study. Interested school principals were followed up telephonically to schedule the interviews. Permission to recruit grade 4 to 7 teachers was sought from participating principals who were sent project information materials and consent forms for distribution to potential teacher participants, via email. FGDs were scheduled with willing teacher participants. Figure 1 describes the sampling and recruitment strategy. Data were collected directly from all participant principals and teachers, with no need for language interpretation.

### Data collection

Appointments were scheduled during the school day to allow principals to attend after-school meetings and professional development events. In-person FGDs were held with teachers to stimulate dynamic conversations leading to discovery, exploration, and depth about the topic. FGDs with schoolteachers were conducted either in the school hall, staff room or library where there would be no disturbances and they could feel comfortable sharing their thoughts and perspectives. FGDs with teachers were scheduled at the end of the school day after they had completed their lesson preparation for the following day. Although non-participants were allowed to attend the FGDs on request, no non-participants attended. Data collection took place between Term 3 of 2017 and Term 1 of 2018 apart from Term 4 of 2018 so as not to disturb the end-of-year examination preparation.

The interview scheduled was piloted during an IDI with a school principal, during which the technical and logistical aspects of conducting future IDIs were assessed. The IDI was 32 min long and had adequate audio quality. The analysis of the transcript revealed that the interview schedule was adequate to address the study objectives. Data from the pilot interview were included in the final analysis. All interviews were conducted by the first interviewer (DF) although a second interviewer (QL) attended a sample of the interviews to monitor consistency and coherence. All IDIs and FGDs were once-off, with no follow-ups required.

DF is a male physiotherapist who underwent a five-day short course in qualitative research methodology that included three written assignments and examination prior to the start of the project. DF has a master’s degree in physiotherapy and this study formed part of his larger doctoral research project. Co-author and doctoral supervisor, QL, is an experienced researcher including qualitative research designs. Neither author had prior relationship with the study participants other than during the recruiting process where participants were informed about the nature of the research project.

The interview schedule (Appendix 3) was co-designed by the authors and aimed at understanding the factors that contribute to learners’ movement during class time. Probing questions were employed for clarification and drawing out respondents’ deeper reflections.

### Data management and analysis

Interviews were recorded with a digital Dictaphone. A professional transcription service was used to produce the interview transcriptions. Transcripts and interview audio files were stored in a password protected project folder in the qualitative research analysis software, Atlas.Ti 9*. One author (DF) listened back to all the audio files to correct any discrepancies within the transcription text. DF conducted the process of correcting transcripts which facilitated immersion in the data. The first 3 interviews coded by the PI were then reviewed by QL for agreement on the relevance and definition of identified codes. Differences in code relevance and definition were discussed before reaching consensus.

Coded information was systematically grouped to form overarching themes. Larger themes were refined into sub-themes for greater depth of meaning. In instances where further resolution was helpful, categories were created. The final codebook was then applied to the rest of the transcripts.

An inductive analysis of all the data was conducted by the authors. Themes pertaining to factors that determined learners’ movement during class time emerged following systematic application of the developed codebook to the interview and focus group transcripts. The analysis and organization of the data into themes, sub-themes and categories provide a rich presentation of the participants’ responses. Theme development was iteratively discussed and reviewed by the authors until an agreed theme structure emerged to describe the factors that determined learners’ movement during class time.

### Trustworthiness of the process

#### Creibility

Measures to ensure the credibility of the study included triangulation of the data. This was achieved through consulting interview and reflexive notes produced by the PI. Member checking was not conducted as transcripts were corrected through listening to the recordings of the IDIs and FGDs. DF and QL reached consensus on coding and analysis based on a sample of the transcripts. The use of randomly sampling the schools also served to evenly distribute any ‘unknown influences’ that may have affected participant selection within the sample. In addition, regular debriefing sessions with the second interviewer QL, provided a sounding board to test developing ideas and interpretations and to highlight any biases that may have arisen.

#### Transferability

Details of participant schools and their contexts are provided for readers to assess the applicability to other contexts.

#### Dependability

To ensure dependability of the study, the researcher (DF) maintained detailed operational documents of data gathering. The strategy of overlapping IDIs with FGDs provided additional merit to the study dependability. Regular debriefing with the co-author facilitated a reflective appraisal of the effectiveness of the inquiry into the research topic.

#### Confirmability

A detailed description of the methodology is presented to facilitate the confirmability of the study. Regular consultation between DF and QL guided decision points throughout the data collection, analysis, and interpretation process. These consultations were aimed at ensuring that our own biases were not dominating data analysis and interpretation of participants’ responses.

#### Ethical considerations

The most important ethical consideration confronted during the study related to protection of participant data. To that end, a thorough explanation was provided either telephonically or through providing study information and consent forms via email. Researchers’ contact details were provided if participants required additional information. All data generated from the study (either from voice recordings or written transcripts) was stored on a password protected device. In addition, the Atlas.ti project was additionally password protected, providing a two-step protection of participant data. No data generated was linked to individual participants. Written informed consent was obtained from all participants prior to the interviews and focus group discussions. IDIs were conducted with school principals while FGDs were conducted for grade 4 to 7 teachers. School principals and teachers were interviewed separately due to the potential power dynamics between the two groups that could influence responses from participants. Although research participants were not compensated for their time, snacks and beverages were provided for all participants as data collection often coincided with lunch or recess times.

## Results

### Description of participants

Thirteen primary school principals and 24 teachers participated in this study. Participant demographics are described in Table 1.

**Table 1:** Participant characteristics

	Principals (n = 13)	Teachers (n = 24)
**Gender **		
Female	4	19
Male	9	5
**Age range (years)**		
20–29		1
30–39		1
50–60	6	2
**Teaching experience (years)**		1
< 10		1
10–19		2
20–29		
30–39	5	
> 40	1	
**Grades**		
4		11
5		11
6		1
7		1
**School quintiles**		
3	2	
4	5	9
5	6	15

*Not all participants completed demographic data as requested

Data are presented according to the most relevant themes regarding the participants’ perspectives of learners’ in-classroom movement and body position (Table 2). Participant quotes are provided with participant identifiers, namely, whether principal (PR) or teacher (TE), school quintile and gender. Most quotes were made in English. There was one occasion that a bi-lingual participant provided quotes in Afrikaans. DF translated the Afrikaans quote into English. Back translation was used to ensure the meaning of the quotes were not changed, and was verified by QL.

The IDIs with principals ranged duration between 28 and 53 min (average duration 37.77 min). The duration of FGDs with teachers ranged between 27 and 46 min (average duration 35.33 min).

### Study findings

The factors that influence learners’ movement during class time were grouped according to four main themes related to the teacher, the curriculum, the classroom, and the institution (Table 2). These factors were further categorised, providing greater of insight into the prevailing factors and influences on learners’ movement during class time.

**Table 2:** Main study themes, sub-themes, and categories

Themes	Sub-themes	Categories
Teacher factors	Classroom management practice	Skill level
		Pedagogic approach
	Knowledge and beliefs about sitting and movement	Effects on concentration
		Effects on health
	Classroom management style	Strict or lenient
		Teaching focused or learner centered
Curriculum factors	Volume of academic content and administration	Time pressure
	Prescriptive curriculum delivery	Teacher autonomy
Classroom factors	Learner to classroom space ratio	
	Learner to teacher ratio	
	Classroom furniture	Anthropometric mismatching
		Comfort and ergonomic utility
Institutional factors	Institutional expectations	
	Institutional acceptability	

The overall effect of the factors identified result in learners being able to either move more or to be more sedentary during class time. The extent to which these factors are modifiable will influence the extent to which learners’ movement may be increased or not.

### Teacher factors

Teachers principally determine the degree to which learners are allowed to move during class time. Their classroom management practice, knowledge and beliefs about sitting and movement, and management style contribute to how they influence learners’ freedom to move during class time.

### Classroom management practice

The extent to which teachers’ classroom management practice influences learners’ movement in the classroom is determined by their level of classroom management skill and their pedagogic approach to teaching.

#### Skill level

Teachers’ classroom management practices vary dependent on their classroom management skill. Teachers with greater classroom management skill reported being more equipped to maintain learners’ behaviour and discipline. As such, they were less reliant on learners remaining seated during class time, compared to less skilled teachers, who were more inclined to enforce prolonged periods of sitting as a means of controlling learner behaviour.

*If you’re a good classroom manager, nothing* (related to sitting or standing) *can cause problems in a classroom. (PR08, male, quintile 4)*

#### Pedagogic approach

Teachers’ influence on learners’ movement during class time may vary according to their pedagogic approach and the subject being taught. For example, dialogic approaches adopted in certain subjects may require learners to remain seated for the lesson, while employing collaborative approaches in other subjects may require learners to stand around a table in groups.

…that whole movement of children you can say is subject specific as well as educator specific. (TE06, male, quintile 5)

### Knowledge and beliefs about sitting

Teachers’ beliefs about how learners’ sitting behaviour during class effects their health and ability to concentrate influences how much they allow learners to move during class time.

#### Effects on concentration

Teachers’ knowledge and beliefs about the effects of sitting on learners’ ability to concentrate during class may influence their decisions about whether learners remained sitting during class time. While some teachers believed that interrupting prolonged periods of sitting was helpful to stimulate learners’ concentration, some believed that concentration is optimised when learners are sitting and were more likely to insist that they remain seated during class time.

It is proven that when they’re sitting and they’re quiet and they have all the attention on you as the teacher and you do afterwards ask them questions about the particular thing that you did… (TE05, female, quintile 4).

#### Effects on health

Teachers reported that they weighed up the potential health benefit of reduced classroom sitting with the potential associated detrimental educational effects. Their knowledge and beliefs of the effects of sitting and movement on learners’ health influenced how much they allowed learners to move during class time.

…might be more detrimental to education than what it is beneficial for health. (TE06, male, quintile 5)

### Classroom management style

Whether teachers had a more strict or more lenient classroom management style, and whether they were teaching focused or learner centred influenced how much they allow learners to move during class time.

#### Strict vs. lenient

Learners felt more autonomy to get out of their seats during class in the presence of a more lenient teacher. However, they were more inclined to remain seated when a stricter teacher is present. Learners’ movement behaviour is dependent on cues from different teachers as to whether they exercise perceived autonomy to leave their seating and move during class time.

… if a lenient teacher enters the class you will find that some children will stand up and they will walk around nonchalantly and if a strict teacher enter the class they will be confined so the mind is conditioned already and the nature of the educator entering the class. (TE06, male, quintile 5)

#### Teaching focus or learner-centered style

The degree to which teachers are focused on their teaching role may influence how much learners are allowed to move during class time. More learner-centered teachers may consider learners’ needs (to move) and thus allow them more freedom to do so, compared with teachers who are more focused on their role during teaching activities.

…it depends on how we perceive the way learners should be in class, there are teachers that are still, for instance, having those stereotypes that if you are in the classroom you must be in that controlled way of sitting and all that, you don’t have to move,… (PR13, female, quintile 3).

### Curriculum factors

Teachers cite the prescriptive and time-consuming nature of the curriculum as a barrier to how much consideration they have about learners’ classroom movement.

### Volume of academic content and administration

The time demands of the curriculum prevented teachers from considering the learners’ holistic needs. Teachers perceived the National Curriculum and Assessment Policy Statement (CAPS) to be rigid and time intensive. They expressed their perception of the curriculum as being inflexible and creating pressure to focus on teaching and learning activities, leading to a sense that they do not have the capacity to focus on anything else.

The curriculum is too tight for us to still even have time for anything else. (TE05, female, quintile 4)

### Prescriptive curriculum delivery

Teachers may be conflicted by the demands of their educator roles regarding the curriculum delivery and the potentially negative effects of prolonged in-classroom sitting. While they described the need to be aware of learners’ health needs, they were unable to act on their concerns due to the demands of delivering the curriculum.

Our work has to be done, books has to be marked, assessments have to be done and marked and moderated and all of those so we don’t have time. We can’t worry about their spines because we are worried about what they’re learning. (TE05, female, quintile 4)

### Classroom factors

The context of the classroom, namely the amount of available space to move, the ratio of number of learners to teacher and the utility of the classroom furniture influences learners’ classroom movement during class time.

### Learner to classroom space ratio

Teachers report a barrier to offering learners the opportunity to leave their seats for a stretch break during prolonged bouts of sitting. Due to the high numbers of learners per class, there is insufficient space to do so.

It impacts the child, because [die kind kannie eers op staan en move nie] [ **translated the child cannot even stand up and move].** He can’t get up and just stretch his legs. (PR05, male, quintile 5)

### Learner to teacher ratio

Given the high number of learners per teacher, teachers are reluctant to allow learners to leave their seats and move freely around the classroom during class time for the risk of losing control of classroom discipline. As such, teachers are more likely to insist that learners remain seated during class time.

… you also cannot allow them to move around also, because you have 40 in the class…. It’s crowd control, it’s chaos if they have to move around, so most of them are sitting… (PR01, male, quintile 4).

### Classroom furniture

Classroom furniture design influences learners’ classroom movement through an interplay of anthropometric mismatching, comfort, and ergonomic utility.

#### Anthropometric mismatching

Learners who have outgrown their classroom furniture result in an anthropometric mismatch. As a result, they become more restricted while sitting and are less able to relieve their discomfort from prolonged bouts of sitting.

…my learners are bigger now. They’ve got the same desk as Grade 7, it’s cutting into their skin, especially the legs. (TE 02, female quintile 5)

#### Comfort and ergonomic utility

Uncomfortable classroom furniture impairs learners’ ability to focus and results in increased fidgeting. Teachers perceive learners’ fidgeting as something they want to minimize through improved classroom furniture design to increase comfort and encourage uninterrupted sitting.

*“"If the chair is inadequate, it’s obviously going to impact on your ability to focus, to concentrate. You will become fidgety so it needs to be something that’s comfortable…(PR07, male, quintile 4)"”*.

### Institutional factors

The school and broader schooling system influences learners’ movement during class. Schools have certain expectations about learners’ movement behaviour during class time. The degree to which teachers encourage learners to move during class depends on how that practice will be accepted by the institution.

### Institutional expectations

Learners and teachers have become institutionalised; learners think that teachers expect them to remain seated during class time. They have developed the idea that complying with these expectations is a display of good behaviour. Similarly, teachers have learned that the expectation is that learners should remain seated, and that maintaining learners in their seats during class is expected of them.

But they’ve been in the system for long enough to know that this is what the teachers wants, the teacher wants you to be seated at all times so even if they’re in discomfort or whatever, both of them just manage themselves because they know that’s our expectation. (TE02, female, quintile 5)

### Institutional acceptability

Teachers’ classroom management (and by extension, learners’ movement during class) is influenced by the school’s level of acceptability of teaching practice that promotes learners’ movement during class time. Teachers in institutions that do not accept teaching practice that encourages learners to move during class time are less likely to engage in those practice, resulting in learners more likely being confined to being seated.

But that is what they will call a mad teacher, because I can tell you, if you are four colleagues, you alone do it, and the others don’t see the benefit of it, then you’re crazy. (PR05, male, quintile 5)

## Discussion

While previous studies have explored teachers’ perspectives of classroom-based physical activity interventions [24, 31], this study is the first to explore the factors that influence learners’ movement during the class under usual conditions, and the first in South Africa. Greater understanding of these factors will assist in the implementation of contextualised measures aimed at integrating movement strategies into classrooms to reduce the harmful effects of prolonged classroom sitting. Our study identifies the influence of the teacher, the curriculum, the classroom, and the institution as the main factors that influence in-classroom movement and position of learners. While each of these factors affect learners directly, it is likely that the interplay between them acts to reduce their movement and increase their sedentariness during class time.

### Teacher factors

Teachers are driven by their core mandate i.e., the effective delivery of the curriculum. To achieve that goal, teachers make decisions that they deem will create the optimal classroom conditions for teaching and learning. Teachers’ preferred strategy is ensuring that learners remain seated during class time. Given the evidence of high levels of sitting in class from other studies, this strategy appears to be favoured in other countries as well [32–35]. The preference for learners to remain seated during class may stem from teachers’ training and traditional beliefs that are reinforced by studies that focus on how different classroom seating arrangements affect learner engagement [36–38].

Contrary to current teaching practice, there is emerging evidence that pupils’ concentration and engagement with cognitive tasks may be enhanced by interrupting prolonged sitting with bouts of physical activity and standing [39, 40]. Moreover, more sitting time has been associated with higher lapses in concentration [18]. Previously held notions that learners should remain seated for learning is challenged by the evidence of the benefits of in-classroom movement on health and learning. To translate the emerging evidence into practice, further investigation into strategies to challenge and change current teacher practices in the classroom regarding movement is needed. Ironically, educators’ teaching practices may be undermining their core aim to effectively deliver the curriculum.

A reported negative consequence of prolonged sitting is fidgeting. This phenomenon was also reported in a study on the effects of prolonged sitting on the ability to maintain attention in class [41]. Although all the teachers in our study assumed that learning and curriculum delivery is optimal while learners are seated, they acknowledged that learners cannot tolerate sitting for prolonged periods. Fidgeting increases with time and may in part be either a response to inattentiveness or a way to combat it [42]. Educators noticed that learners became more fidgety in response to becoming tired of sitting; they perceived it to be disruptive behaviour that hampers optimal learning and interferes with the learning of others in the class. It is possible that prolonged sitting contributes to waning arousal associated with inattentiveness and that in-classroom movement may be a defence against it. It is recommended that teacher in-service training highlight the shortcomings of the teaching practice that mandates sitting as a means of optimizing the learning environment.

The teachers who attended in-service training on movement integration strategies are familiar with its potential benefits to learners’ attention and general health and wellness. Despite knowing the potential benefits of increased learner movement during class time, they did not always find it practical to implement due to the higher learner to classroom space ratio. Their concern was that increasing learners’ movement during class time would result in losing control of the classroom, particularly for less skilled teachers. This is mirrors findings by published reports on the perceptions of integrating movement into the classroom [21, 22]. Dinkel et al. [19] suggests that teacher training focused more on the behaviour control benefits of classroom movement integration strategies would result in teachers continuing to implement them. Moreover, Routen et al. [24] recommend that classroom movement integration training should be ongoing, target expansion of teachers’ skills and include broader school and policy support to facilitate use of movement strategies. This may increase institutional acceptability of in-classroom movement strategies and reverse institutional expectations that learners should remain seated during class time.

Teachers’ in-service training on learners’ movement in class must move beyond raising awareness of its benefits to health, well-being, and learners’ attention, and focus on the classroom management benefits of controlling learner behaviour. Furthermore, a more holistic school approach including school management and policy to support teachers’ efforts to implement in-classroom movement strategies is required to make an impact on the health of learners.

### Classroom factors

The high ratio of learners to classroom space and the poor ergonomic utility of classroom furniture was cited as a reason for learners being seated and is a barrier to learners’ movement during class time. Teachers who perceived that their classrooms were small were likely to abandon attempts to create a dynamic classroom environment that encouraged movement. This has also been cited as a barrier for formal movement integration programmes that require learners to leave their desks and compete for open floor space [43]. As increasing the size of the classroom is not feasible, a way to increase learners’ movement during routine classroom time is by allowing learners to stand up during collaborative learning activities [44].

Allowing learners to stand up while working on independent tasks, and to move during collaborative learning activities, oral presentations, and reading tasks may provide opportunities for relief from mismatched, uncomfortable classroom furniture and small increases movement during class time without having learners compete for limited floor space. The size, weight, and bulkiness of traditional classroom furniture, however, prohibits teachers from arranging the desks to create collaborative workspaces that encourage in-classroom movement. Furthermore, traditional desks have a set desk height suitable for sitting that is not conducive for use while standing up [45]. Sit-stand desks encourage in-classroom movement by allowing learners to transition between sitting and standing while completing set learning activities. More recently, there is growing evidence of the efficacy of standing desks to facilitate longer bouts of classroom standing time and interrupt prolonged sitting bouts [17, 45, 46]. Sit-stand desks may be a practical solution to the barrier of insufficient space due to small class sizes, high numbers of learners in class and inflexible traditional classroom furniture. In a complex country such as South Africa, high-level support and the design of a new classroom furniture policy will be needed if alternatives such as sit-stand desks are to become an option in local schools.

To our knowledge, this is the first study to explore the factors that influence primary school learners’ movement and position during class time during usual conditions. Despite several teacher and structural factors having been identified in internationally published reports, contextually appropriate interventions to increase learners’ classroom movement must be created. This study is strengthened by the inclusion of diverse study participants. Given the social-economic inequality in South African society, including participants from across the socio-economic spectrum ensures that the study findings are more generalisable. The study sample included teachers and principals but did not include learners. Although school principals do a limited amount of teaching, their primary role is one of school administration. It is possible that their responses during the interviews relied on recalling their experiences from when they were primarily teaching compared to teachers’ responses who were still currently teaching. Future studies may compare the responses from teachers and principals or select a sample of either one or the other. The response from participants about their demographic information was limited and impacts on the generalisability of the study findings.

Implications for future practice and policy.

Compared to other countries dealing with the increasing burden of non-communicable disease and spinal health, very little health promotion addressing these health conditions has taken place in the South African school setting. There is a long history of school-based interventions to reduce SB and promote spinal health in other countries, yet it’s in its infancy in South Africa. Given the strategic potential to impact population health outcomes, greater attention should be placed on understanding the contextual factors within the school system to facilitate health promotion implementation strategies in the school setting without hindering the primary objectives of educators. Despite long standing policy to foster interdepartmental collaboration between the departments of health and education at national policy level [47], grassroots structures and networks must be prioritised to advance the agenda of this collaboration. In short, teachers and health practitioners must organise to combine efforts in addressing the health of school learners.

## Conclusion

This study explored the perspectives of South African educators on learners’ in-classroom movement and position. Educators preferred learners to remain seated as that was considered the most effective way of delivering the curriculum. However, when learners were required to sit for prolonged periods, educators became aware that learners’ attention and concentration began to wane, resulting in disruptive behaviour such as fidgeting. The evidence of the benefits of interrupting prolonged sitting with light movement, such as standing rather than sitting on learner health and classroom control, has not translated into teaching practice. In-service teacher training informed by this evidence is needed to assist teachers in making the classroom more conducive for learning. School administration and policy should support teachers in implementing evidence informed in-classroom movement strategies. Small class sizes with high numbers of learners per class and classroom furniture also hamper the adoption of in-classroom movement strategies. High level policy and political support is needed to challenge and change current classroom practices to promote the health and well-being of learners.

### Electronic supplementary material

Below is the link to the electronic supplementary material.

**Supplementary Material 1:** Participant information and Consent form

**Supplementary Material 2:** Indicative discussion schedule

**Supplementary Material 3:** Project information sheet

## Data Availability

The datasets used and/or analysed during this study are not publicly available due to their nature as qualitative data but are available on request from the corresponding author.
