# Supplementary material for: Primary school learners’ movement during class time: perceptions of educators in the Western Cape, South Africa
Source: BMC Public Health. 2023 Dec 13;23:2501. doi: 10.1186/s12889-023-17428-3 (PMC10720100; doi:10.1186/s12889-023-17428-3)
Supplement: Supplementary file 1 — Supplementary Material 1: Participant information and Consent form [file 12889_2023_17428_MOESM1_ESM.docx]

PARTICIPANT INFORMATION LEAFLET AND CONSENT FORM

TITLE OF THE RESEARCH PROJECT: Sedentariness and back health in Western Cape school learners: a qualitative study

Ethics reference number: S17/08/130

PARTICIPANT FORM: Feasibility_interview_consent_principalteacher

## PARTICIPANT REFERENCE NUMBER:

PRINCIPAL INVESTIGATOR: Mr Dominic Fisher Supervisor: Prof Quinette Louw

ADDRESS: Stellenbosch University, Faculty of Medicine and Health Sciences, Division of Physiotherapy, PO Box 241

## CONTACT NUMBER:

021 938 9667/ 073 304 8496

You are being invited to take part in a research project. Please take some time to read the information presented here, which will explain the details of this project. Please ask the study staff any questions about any part of this project that you do not fully understand. It is very important that you are fully satisfied that you clearly understand what this research entails and how you could be involved. Also, your participation is entirely voluntary, and you are free to decline to participate. If you say no, this will not affect you negatively in any way whatsoever. You are also free to withdraw from the study at any point, even if you do agree to take part.

This study has been approved by the Health Research Ethics Committee at Stellenbosch University and will be conducted according to the ethical guidelines and principles of the international Declaration of Helsinki, South African Guidelines for Good Clinical Practice and the Medical Research Council (MRC) Ethical Guidelines for Research.

## What is this research study all about?

- More and more information is available about the effects of spending long periods of sitting affects our health. Sitting for too long may negatively affect our health in at least two ways, it can be harmful to our backs and it can affect the way the body cells work. Sitting for too long may lead to becoming overweight, developing diabetes and other health problems. School furniture and health education videos can help learners lessen the amount of time they spend sitting and can promote good back health.
- The aim of this research project is to obtain the perspectives of school staff on the use of the new classroom furniture and health education program. Furthermore, the researcher aims to obtain the perspective and feedback from school staff about conducting an intervention study based in the classroom.
- Participants will be asked to participate in an individual interview. All interviews will be recorded on a Dictaphone. The researcher will conduct the interview

according to a schedule of prepared standardised questions. Interviews are estimated to vary in duration from 30 to 45 minutes. The interview will then be transcribed from the audio file and analysed. Participants will then receive a copy of the transcription for validation. Analysis will only resume after the transcription is validated by the participants.

## Why have you been invited to participate?

- Very few studies consisting adapted classroom furniture and a classroom-based health education have been conducted. Obtaining the perspectives and feedback of the teacher and principal experience will provide valuable information that will inform future studies and contribute to their future success. The researcher appreciates that school staff are key stakeholders and are best placed to provide expert input into the planning of proposed classroom-based intervention.

## What will your responsibilities be?

You will be required to participate in 1 interview of the intervention study that your class is participating in. You will be asked to respond to a schedule of questions in an open and honest fashion.

## Will you benefit from taking part in this research?

There are no direct benefits to you but you by agreeing to participate, you will be able to be providing valuable feedback and information that will contribute to the success of future classroom-based studies.

## Are there in risks involved in your taking part in this research?

No foreseeable risks involved in participation in the study.

## If you do not agree to take part, what alternatives do you have?

Not applicable.

## Who will have access to your study records?

All the recordings from the interviews will be transcribed. The taped recordings will be destroyed on completion of the project, while transcriptions will be securely stored at Stellenbosch University for a maximum period of five years. Only the researcher will have access to the records. There will be no means that you will be identified through the audio tape or the written document. The recorded information will be analysed and may be used in the writing of research articles or for writing up the research project thesis.

## What will happen in the unlikely event of some form injury occurring as a direct result of your taking part in this research study?

No foreseeable risks involved in participation in the study.

## Will you be paid to take part in this study and are there any costs involved?

No you will not be paid to take part in the study but your transport and meal costs will be covered for each study visit. There will be no costs involved for you, if you do take part.

## Is there any thing else that you should know or do?

- You can contact Mr Dominic Fisher at tel 021 938 9667 or 073 3048496 if you have any further queries or encounter any problems.
- You can contact the Health Research Ethics Committee at 021-938 9207 if you have any concerns or complaints that have not been adequately addressed by your study doctor.
- You will receive a copy of this information and consent form for your own records.

# Declaration by participant

By signing below, I …………………………………..…………. agree to take part in a research study entitled ‘Sedentariness and back health in Western Cape school learners: a qualitative study’.

I declare that:

- I have read or had read to me this information and consent form and it is written in a language with which I am fluent and comfortable.
- I have had a chance to ask questions and all my questions have been adequately answered.
- I understand that taking part in this study is voluntary and I have not been pressurised to take part.
- I may choose to leave the study at any time and will not be penalised or prejudiced in any way.
- I may be asked to leave the study before it has finished, if the study doctor or researcher feels it is in my best interests, or if I do not follow the study plan, as agreed to.
- I agree to having the interview audio recorded

| YES | NO |
| --- | --- |

Signed at (place) ......................…........…………….. on (date) …………....……….. .

.............................................................. ............................................................

Signature of participant Signature of witness

# Declaration by investigator

I, Dominic Fisher, declare that:

- I explained the information in this document to …………………………………..
- I encouraged him/her to ask questions and took adequate time to answer them.
- I am satisfied that he/she adequately understands all aspects of the research, as discussed above
- I did/did not use a interpreter. (If a interpreter is used then the interpreter must sign the declaration below.

Signed at (place) ......................…........…………….. on (date) …………....……….. .

.............................................................. ............................................................

Signature of investigator Signature of witness

# Declaration by interpreter

I (name) declare that:

- I assisted the investigator (Dominic Fisher) to explain the information in this document to (name of participant) ……………..……………………………..

using the language medium of Afrikaans/Xhosa.

- We encouraged him/her to ask questions and took adequate time to answer them.
- I conveyed a factually correct version of what was related to me.
- I am satisfied that the participant fully understands the content of this informed consent document and has had all his/her question satisfactorily answered.

Signed at (place) ......................…........…………….. on (date) …………....………………..

.............................................................. ............................................................

## Signature of interpreter Signature of witness
