# Supplementary material for: Primary school learners’ movement during class time: perceptions of educators in the Western Cape, South Africa
Source: BMC Public Health. 2023 Dec 13;23:2501. doi: 10.1186/s12889-023-17428-3 (PMC10720100; doi:10.1186/s12889-023-17428-3)
Supplement: Supplementary file 2 — Supplementary Material 2: Indicative discussion schedule [file 12889_2023_17428_MOESM2_ESM.docx]

## Discussion Schedule for IDI’s & FGD’s

Participants Sub-group: Principal / Teachers

Grade: ǀ__ǀ__ǀ

Audio File #: ǀ__ǀ__ǀ__ǀ Date: / /

Introduction

- General purpose of study
- Aims of the interview
- Thanks for participation
- What will happen with collected data
- Consent given. May stop interview at any time

1. How would you describe the in-classroom movement of learners during the school day?
2. How aware are you about the in-classroom movement and body position of learners?
3. How does the learners’ environment influence their in-classroom movement and body position during class?
4. How do you influence the in-classroom movement and body position during class time?
5. What is your perspective on in-classroom movement?
6. What are the effects of in-classroom movement on learners (academic and health)?

- Perceived benefits / harms

1. Do you incorporate movement during class?
2. Do you have any final comments about the topics discussed today?

Thank you for your participation.
