# Supplementary material for: Primary school learners’ movement during class time: perceptions of educators in the Western Cape, South Africa
Source: BMC Public Health. 2023 Dec 13;23:2501. doi: 10.1186/s12889-023-17428-3 (PMC10720100; doi:10.1186/s12889-023-17428-3)
Supplement: Supplementary file 3 — Supplementary Material 3: Project information sheet [file 12889_2023_17428_MOESM3_ESM.docx]

Project information

Classroom sedentariness and back health prevention: a qualitative study

A lifestyle of sedentary behaviour may result in the development of two non-communicable conditions, such as metabolic syndrome and poor back health. Sedentariness refers to any waking behaviour which involves energy expenditure less than or equal to 1.5 metabolic equivalent units (METs) while sitting, lying down or reclining. Even when the guidelines for physical activity are met, sedentariness remains an independent risk factor for the development of non-communicable diseases (NCDs), and musculoskeletal disorders. Excess sitting has been associated with increasing incidence of NCDs. Furthermore, prolonged sitting adversely affects back pain.

The aetiology of back pain is multifactorial; poor postural alignment and postural dynamism are common risk factors of back pain. Back pain is a significant contributor to absence of health in scholars, thus school furniture should facilitate postural dynamism during sitting, regular sit-stand transitions and optimal postural alignment to reduce sedentariness and consequently back pain and NCD risk in the longer term. Only five intervention studies to address sedentariness in primary school children are currently available. These studies show promising effectiveness for sit-stand classroom furniture to address sedentariness. Currently, no back health intervention studies have addressed sedentariness, although it is an important risk factor.

In low and middle income countries, preventative back health strategies should be integrated into programs and policies for NCDs to spare constrained health care resources. To our knowledge, no studies have addressed sedentariness by assessing the effect of a multi- modal approach for sedentariness to promote back health and reduce the risk of developing NCDs.

Therefore, the aim of this project is to provide new information about the feasibility of a classroom-based intervention aimed at reducing sedentariness in primary school learners.

# QUALITATIVE DESIGN

Objectives:

- Identify perspectives of sedentary behaviour and back health amongst WCED officials, school principals and teachers, learners and parents through in-depth interviews and focus group discussions.
- Obtain the perceptions, attitudes and feedback of the proposed classroom-based intervention from WCED officials, school principals and teachers, learners and parents; identify perceived facilitators and barriers to the classroom-based intervention through in-depth interviews and focus group discussions.

# PHASE 2 OUTCOME:

Modification of proposed intervention based on the feedback and findings of the qualitative study.
